# A propeptide toolbox for secretion optimization of *Flavobacterium meningosepticum* endopeptidase in *Lactococcus lactis*

**DOI:** 10.1186/s12934-017-0836-0

**Published:** 2017-12-05

**Authors:** Pei Yu Lim, Lee Ling Tan, Dave Siak-Wei Ow, Fong T. Wong

**Affiliations:** 1 Microbial Cell Group, Bioprocessing Technology Institute, Agency for Science, Technology and Research (A*STAR), 20 Biopolis Way, #06-01 Centros, Singapore, 138668 Singapore; 2 Molecular Engineering Lab, Biomedical Sciences Institutes, A*STAR, 61 Biopolis Drive, Singapore, 138673 Singapore

**Keywords:** *Flavobacterium meningosepticum* prolyl endopeptidase, Lactic acid bacteria, *Lactococcus lactis*, Secretion propeptide, Recombinant protein expression

## Abstract

**Background:**

Lactic acid bacteria are a family of “generally regarded as safe” organisms traditionally used for food fermentation. In recent years, they have started to emerge as potential chassis for heterologous protein production. And more recently, due to their beneficial properties in the gut, they have been examined as potential candidates for mucosal delivery vectors, especially for acid-sensitive enzymes. One such application would be the delivery of gluten-digesting endopeptidases for the treatment of celiac disease. To facilitate these applications, an efficient recombinant protein expression toolbox is required, especially for recombinant protein secretion. While current tools for enhancing protein secretion consist mainly of signal peptides, secretion propeptides have also been observed to play a crucial role for protein secretion and improved yields.

**Results:**

To expand the propeptide library for secretion optimization, we have mined and characterized three naturally occurring propeptides from the sequenced genomes of 109 *Lactococcus* species. These newly-mined propeptides were introduced after the N-terminal USP45 secretion signal to characterize and compare their effects on the secretion of *Escherichia coli* thioredoxin (TRX) and *Flavobacterium meningosepticum* prolyl endopeptidase (Fm PEP) in *Lactococcus lactis* NZ9000. All three propeptides, along with the positive control LEISSTCDA, improved volumetric secretion yields by 1.4–2.3-folds. However, enhancement of secretion yield is dependent on protein of interest. For TRX, the optimal combination of USP45 signal peptide and LEISSTCDA produced a 2.3-fold increase in secretion yields. Whilst for Fm PEP, propeptide 1 with USP45 signal peptide improved volumetric secretion yields by 2.2-fold compared to a 1.4-fold increase by LEISSTCDA. Similar trends in Fm PEP activity and protein yield also demonstrated minimal effect of the negative charged propeptides on PEP activity and thus folding.

**Conclusions:**

Overall, we have characterized three new propeptides for use in *L. lactis* secretion optimization. From success of these propeptides for improvement of secretion yields, we anticipate this collection to be valuable to heterologous protein secretion optimisation in lactic acid bacteria. We have also demonstrated for the first time, secretion of Fm PEP in *L. lactis* for potential use as a therapy agent in celiac disease.

**Electronic supplementary material:**

The online version of this article (10.1186/s12934-017-0836-0) contains supplementary material, which is available to authorized users.

## Background

Lactic acid bacteria (LAB) are a promising family of food-grade organisms for heterologous protein production due to its Generally Regarded as Safe (GRAS) status. Representative genera of LAB include *Lactobacillus*, *Bifidobacterium*, *Lactococcus*, *Aerococcus*, *Leuconostoc*, *Oenococcus* and *Pediococcus* [1]. Traditionally, LAB were utilized in food as starter cultures for fermentation and as probiotics [2]. Studies of LAB and host interactions have also associated LAB directly with cellular activities of the gut, such as pathogen control, immunostimulation and maintaining a healthy microflora [3, 4]. Combined with their traditional roles in food fermentation, beneficial gut properties, and resistance to harsh gut conditions, additional advantages such as LAB’s ability to secrete recombinant proteins while possessing fewer proteases, compared to traditional workhorses, such as *Escherichia coli* and *Bacillus subtilis*, have made them attractive targets for recombinant cell factories and live vectors for delivery of therapeutic molecules in the gut [5].

Celiac disease is an inflammatory autoimmune disorder of the small intestine arising from intolerance to gluten in food. Traditional treatment of celiac disease mainly consists of dietary restrictions, however even traces of gluten contaminants can be immunogenic and result in detrimental consequences over time. A recent promising development of oral therapy for celiac disease, using food grade prolyl endopeptidase (PEP) to break down contaminant gluten, have sparked interest into exploring non-dietary alternatives using mucosal enzyme delivery vectors, such as LAB. Delivery of digestive enzymes to breakdown gluten have been investigated for predigested gluten and in situ gastrointestinal lumen treatment. In the latter, oral therapy using combination of acid-resistant food grade digestive enzymes have recently entered phase III trials for celiac disease treatment [6]. However, for acid sensitive PEPs, delivery efficiency and the ability of delivery vectors to survive through the gastrointestinal tract would have to be examined and optimized. Previously, secretion of *Myxococcus xanthus* prolyl endopeptidase (Mx PEP) has been explored successfully in *Lactobacillus casei* [7] for the digestion of the 33-mer gliadin-derived epitopes in an in vitro small intestine model. However, in an oral administration study in rats, Mx PEP did not shown PEP-specific cleavage compared to the *Flavobacterium meningosepticum* prolyl endopeptidase (Fm PEP) [8]. Hence, demonstration of secreted and functional Fm PEP from LAB would be a significant step for the prospective treatment of celiac disease.

To accomplish these goals, tools that enable efficient recombinant protein expression in LAB, are essential. Although genomics, metabolic pathways and genetic toolboxes, consisting of cloning vectors, mutagenesis systems [9], have been well developed and studied, tools for engineering the LAB recombinant protein cassette for protein secretion focus mainly on vector [10–12], strain optimisation [13] and signal peptides [14–16]. The latter focus mainly on screening and mutagenesis of signal peptides, in addition to the commonly used USP45, to create signal peptide libraries [14, 17]. Less intensively investigated is the development of secretion propeptides for secretion efficiencies and thus yield enhancement [9]. The main propeptide currently in use, LEISSTCDA, was first created for secretion of *Staphylococcus aureus* nuclease and then subsequently utilized for other recombinant proteins in LAB. Although, LEISSTCDA has been used in various studies to increase secretion yields [18], it is not an universal enhancer of heterologous secretion, for example, in the case of an anti-freeze protein, a reduction in secretion was observed instead [19]. Further mutagenesis within LEISSCTDA did not yield improved propeptides [20]. This suggests a larger secretion propeptide library would be critical in further optimizing secretion yields of different recombinant proteins in LAB.

In this study, we exploited advancement in genomics and availability of sequenced LAB genomes assemblies to mine for secretion propeptides. Three unique propeptide sequences were identified and they were evaluated in parallel with commonly used LEISSCTDA on secretion of two recombinant proteins in *Lactococcus lactis*. The newly mined propeptides were first characterized using secretion of recombinant *E. coli* thioredoxin (TRX), which has been used extensively as a solubility partner for recombinant protein expression in *E. coli* [21, 22] and more recently, utilized as an intracellular solubility partner in *L. lactis* [23]. Next they were then applied for the secretion of 78 kDa Fm PEP. This is the first instance of Fm PEP secretion and its optimization in *L. lactis*.

## Results

### Mining of propeptides

**Fig. 1:**
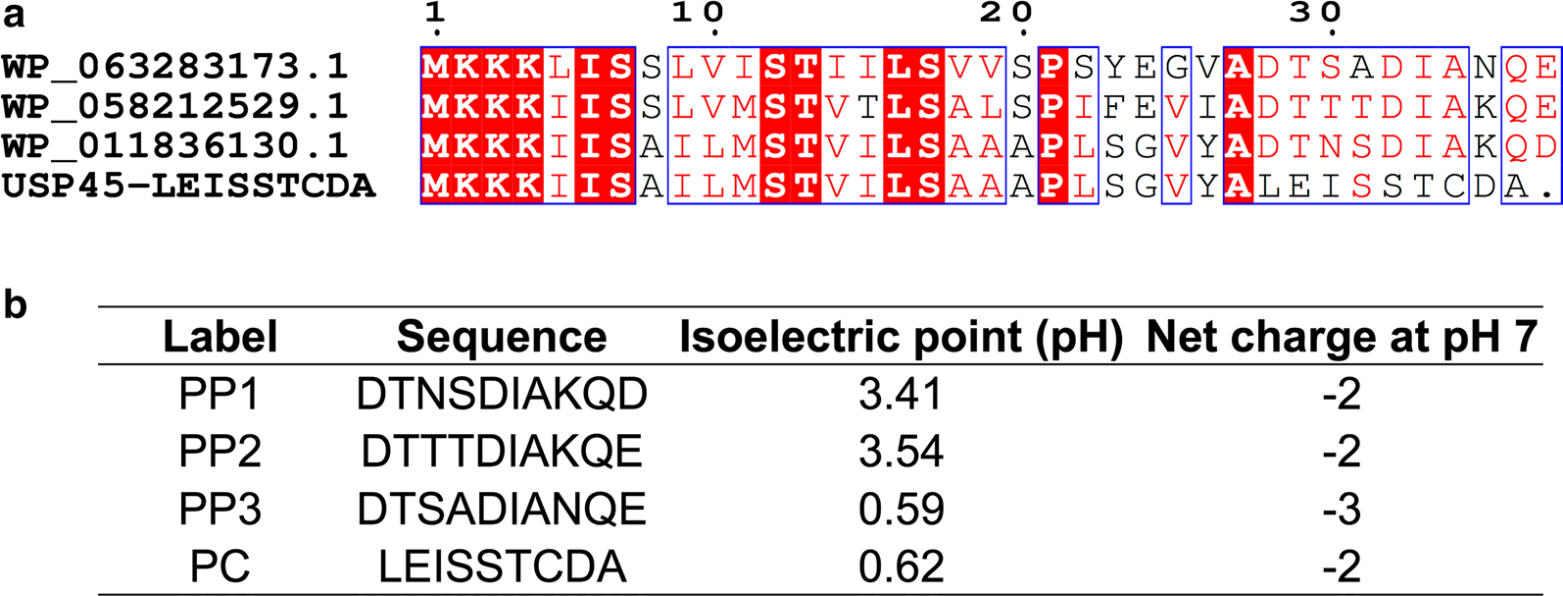
Mining of propeptides from sequenced *Lactococcus* genome assemblies. **a** Alignment of native *Lactococcus* proteins with USP45-LEISSTCDA sequence. Alignment was created in ESPript 3.0 [31]. **b** Comparison of isoelectric points and net charges of the three propeptides (PP1–PP3) and positive control LEISSTCDA (PC) propeptide

We first chose a reference signal peptide, the naturally occurring signal peptide from *L. lactis* secreted protein of unknown function (USP45 SP). Besides being the currently most utilized secretion signal peptide for *L. lactis*, USP45 SP has also been shown to be more efficient over other natural signal peptides (SP310 [24]) or mutated libraries (USP45MT11 [14], SP310mut2 [17]). To mine for secretion propeptides, the amino acid sequence of USP45 SP (Accession Code: ABY84357) was used to blast against 109 deposited assemblies of *Lactococcus* species (accessed October 18, 2016). From the BlastP search, three propeptide sequences, which were most representative of the results, were identified (Fig. 1a). The estimated frequency of these propeptides from the BLAST search (not accounting for highly identical sequences); PP1:PP2:PP3 are 59:3:9. The isoelectric point of these propeptides ranged from 0.6 to 3.5, compared to 0.6 for our positive control propeptide LEISSTCDA. All three propeptides have a net charge of − 2 to − 3 at pH 7 (Fig. 1b). The negative charges of the propeptides are contributed by the multiple aspartic acid and glutamic acid residues in the sequences. These observations also correspond to the previous finding that negative charge of LEISSTCDA plays a significant role in increasing secretion efficiencies [18]. Henceforth for simplicity, the three data-mined propeptides will be labelled as PP1–PP3 (Fig. 1b).

### TRX secretion and optimization

**Fig. 2:**
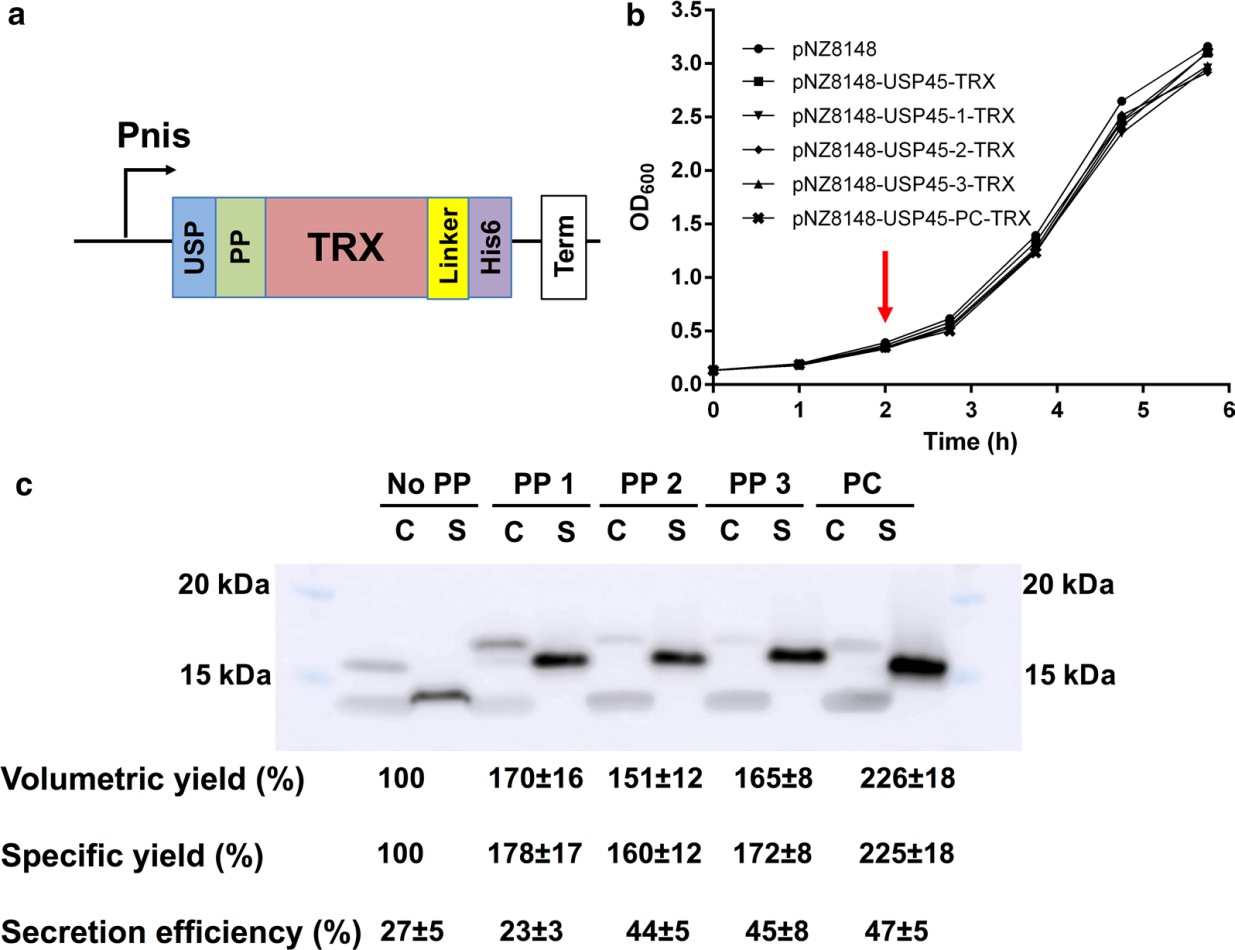
Secretion of TRX in NZ9000. **a** Expression construct for secreted TRX, USP: USP45, PP: propeptide, Linker: flexible linker with TEV cleavage site, His6: HisTag, Term: terminator. **b** Growth curves for pNZ8148 vector only, USP45-TRX, USP45-PP1-, -PP2-, -PP3-, -PC-TRX. Induction is indicated by a red arrow. **c** Representative Western blot of cell lysate (C) and secreted (S) fractions. Cell lysate and secreted fractions were concentrated 3 and 25 times respectively and 2 μL of each was loaded onto the gel. The lowest conserved band observed in the cell lysate fraction is a non-specific binding artefact (Additional file 1: Figure S1). % protein yields, with reference to USP45-TRX, and secretion efficiencies of the various TRX constructs are given below. These were calculated based on densitometry and technical triplicates

As an initial evaluation of these propeptides, we utilized the soluble 15 kDa *E. coli* TRX as our reporter protein. The glycine–serine linked protein expression cassette (Fig. 2a) consisted of USP45 SP, followed by the propeptide of interest, the N-terminal of the codon-optimized TRX gene cassette. The C-terminal of the gene cassette consisted of a glycine–serine–alanine (GSGSGAAA) linker before a TEV cleavage site (ENLYFQG) and a his6-tag (HHHHHH). In our control expression cassette, there are no propeptide and only one GS linker between USP45 SP and TRX. The protein expression cassette was introduced into nisin-inducible pNZ8148 via DNA assembly.

The final constructs were transformed into NZ9000 and grown at 30 °C. Induction of the culture proceed at OD_600_ = 0.5 with 10 ng/mL nisin (Fig. 2b). After 3 h of expression, his-tagged proteins of the expected sizes (15–19 kDa) were observed (Fig. 2c). Bands on the Western blot, corresponding to full length (17 kDa) USP45-TRX and its truncated (15 kDa) TRX, were observed for USP45-TRX in intracellular and secreted fractions respectively. Similarly, for USP45-propeptide-TRX constructs, bands corresponding to full length (19 kDa) USP45-propeptide TRX and truncated (16 kDa) propeptide-TRX were also observed in intracellular and extracellular fractions respectively. From the observed sizes of the TRX constructs, truncation of the secreted protein is predicted to occur between the signal peptide and propeptides. This is as predicted with SignalP 4.1 [25].

With USP45 SP only, a 27% secretion efficiency for TRX was observed. Whilst upon insertion of propeptides, a maximum of 1.7-fold improvement in secretion efficiency (LEISSTCDA, 47%, Fig. 2c) was observed. With increased secretion efficiencies, there are also corresponding 1.5–2.3-folds increases in both volumetric and specific secretion yields for propeptide containing constructs, compared to without propeptide (Fig. 2c). Overall, PP1–3 were observed to enhance both secretion efficiencies and yields like LEISSTCDA. However, for TRX, the positive control LEISSTCDA still yielded the highest amount of secreted TRX, among the propeptides.

### Expression and secretion of functional Fm PEP

**Fig. 3:**
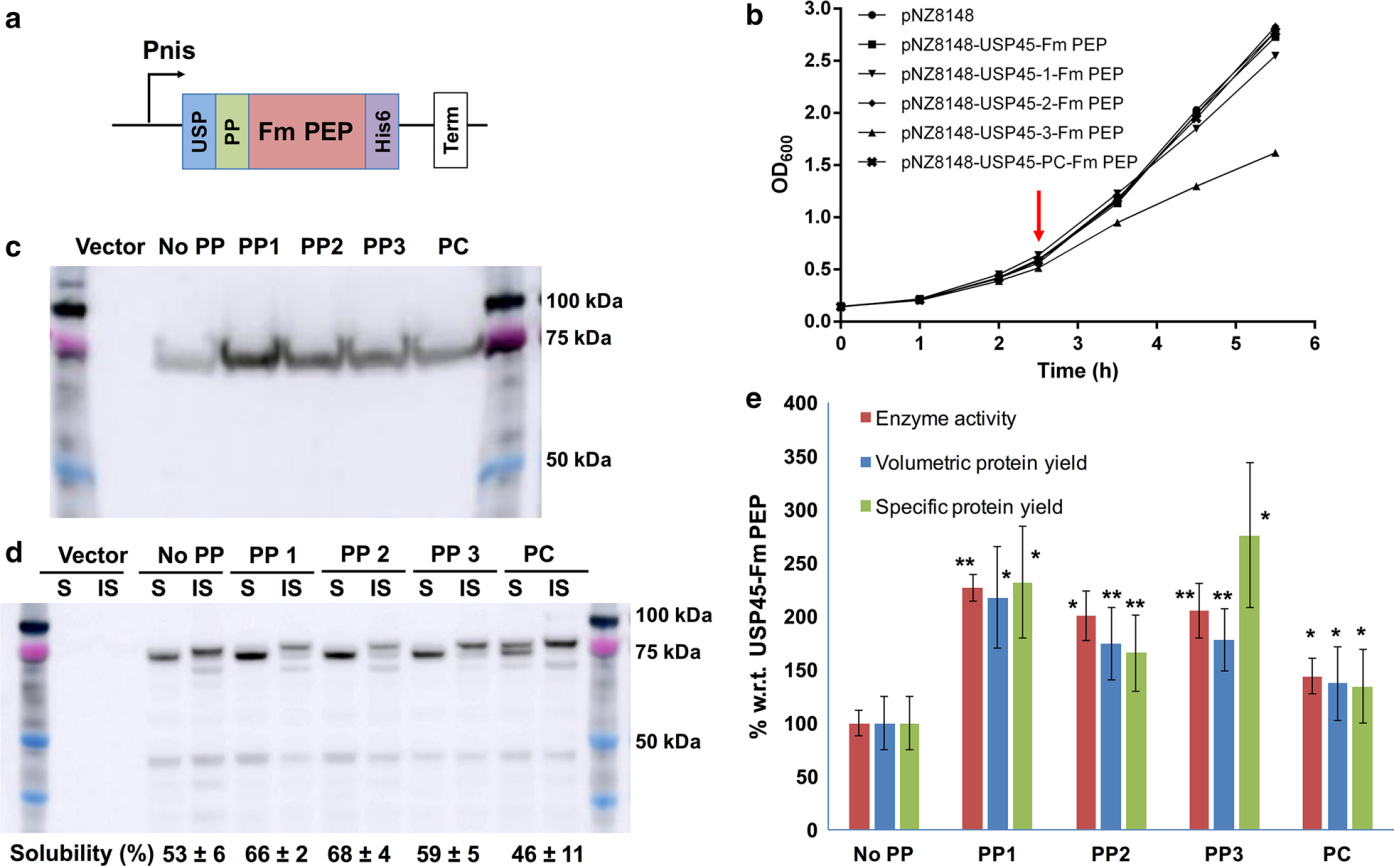
Secretion of Fm PEP in NZ9000. **a** Expression construct for secreted Fm PEP, USP: USP45, PP: propeptide, His6: HisTag, Term: terminator. **b** Growth curves for pNZ8148 vector only, USP45-FmPEP, USP45-PP1-, -PP2-, -PP3-, -PC-FmPEP. Induction is indicated by a red arrow. **c** Comparison of media fractions on a representative Western blot. Vector refers to expression of an empty pNZ8148 vector. **d** Representative Western blot comparison of soluble (S) and insoluble (IS) fractions in the cell lysate. Vector refers to expression of an empty pNZ8148 vector. Cell lysate and secreted fractions were concentrated 3 and 25 times respectively and 2 μL of each was loaded onto the gel. **e** % secretion yields and secretion efficiencies for the various constructs with respect to USP45-Fm PEP. Secretion protein yields are calculated based on densitometry. Enzyme activities were calculated based on Z-gly-pro-4-nitroanilide assay. Biological and technical triplicates were performed for these experiments, significant at *p < 0.05, **p < 0.01. Results are summarized in Additional file 1: Table S1

Next, we examine effects of PP1–3 on the secretion of Fm PEP. The glycine–serine linked protein expression cassette consisted of USP45 SP, followed by the propeptide of interest, the codon-optimized Fm PEP gene cassette and a his6-tag at the C-terminal (Fig. 3a). In our control expression cassette, there are no propeptide and only one GS linker between USP45 SP and Fm PEP. Again, the protein expression cassette was introduced into nisin-inducible pNZ8148 via DNA assembly.

After nisin induction of NZ9000-transformed strains at 30 °C for 3 h (Fig. 3b), both cell lysate and media were analysed by Western blot analysis (Fig. 3c, d). Bands at 75 kDa corresponding to the size of Fm PEP were observed in both cell lysate and media fractions (Fig. 3c, d). Two closely migrated bands, observed near 75 kDa, are predicted to be full length constructs (84 and 83 kDa for with and without propeptide respectively) and truncated Fm PEP (81 and 80 kDa for with and without propeptide respectively) (Fig. 3c, d). Bands of the truncated Fm PEPs were observed in the soluble intracellular fraction, suggesting that the precursors are undergoing cleavage within the cells. In the intracellular fractions, solubility of the proteins range from 46 to 68% of the total intracellular proteins, and from our enzymatic assays, the soluble intracellular proteins were found to be active (Additional file 1: Figure S2). A general increase in volumetric secretion yields (1.4–2.2-folds) was observed with the introduction of the propeptides into the Fm PEP expression cassette (Fig. 3e). When normalized to optical density, increases in specific secretion yields are 2.3, 1.7, 2.8 and 1.3-folds for PP1, PP2, PP3 and LEISSTCDA respectively. The higher specific yield of PP3 is a result of significant reduced growth of the host cell after nisin induction (Fig. 3b).

Comparison of PEP activities in the media fractions also displayed a similar enhancement trend to that of the secretion yields (Fig. 3e). This also suggest that there are minimal effects on Fm PEP activity from insertion of different highly negative charged propeptides at the N-terminus. Comparison between propeptides also indicated that a different preference of propeptide was demonstrated by Fm PEP compared to TRX. For Fm PEP, PP1 produced the best volumetric secretion yield and activity (2.2-fold) whilst the positive control LEISSTCDA only managed a 1.4-fold increase in yield and activity.

Although we demonstrated secretion of active Fm PEP in *L. lactis*, the secretion efficiencies for Fm PEP (estimated 0.9–1.6%) is still significantly lower than that of TRX (23–47%). This could be attributed to the higher solubility and lower molecular weight of TRX compared to Fm PEP. Strikingly, although folding is not expected to take place with the presence of the signal peptide intracellularly, the cell lysate was found to contain soluble, cleaved and functional Fm PEP proteins (Fig. 3d, Additional file 1: Figure S2). These observations suggest that further design optimization could be used to reduce intracellular cleavage and in turn, increase secretion efficiencies.

In examination of growth rates, slower growth were observed in PP1 and PP3 for Fm PEP, especially so for the latter. This decrease in growth rates after induction could be contributed to inability of the cells to handle the additional stress of protein secretion [26, 27]. This also suggests that higher yield can be achieved by optimization of strains and systems for secretion [10–13].

## Discussion

In this study, 3 naturally occurring propeptides were examined in addition to the widely utilized synthetic propeptide LEISSTCDA. The set of 4 propeptides were evaluated using two different recombinant proteins, where the ability to increase secretion yields and efficiencies were demonstrated for all 4 highly negative charged propeptides. However, from characterisation of 2 proteins, it was shown that the optimal propeptide for each protein with USP45 SP is not the same. With TRX, the LEISSTCDA produced the highest secretion yields, although similar increases in secretion efficiencies were observed for PP3, PP2 and LEISSTCDA. With Fm PEP, the highest volumetric yield and secretion efficiency were obtained by PP1. These observations emphasize once again that success of heterologous protein expression and secretion are hard to predict rationally. Interactions of the signal peptides, propeptides and protein of interest, including balancing of translation, secretion and folding pathways, are all contributors to its final secretion yield and efficiency [9]. Subsequently, this highlights the importance of a well-characterized and tested toolbox for rapid screening and optimization of the recombinant protein secretion in LAB.

In our examination of the newly-mined propeptides, we have also demonstrated secretion of functional Fm PEP. Previously, Fm PEP have been examined as a potential PEP candidate for celiac disease treatment, where the enzyme has been shown to be resistant to the acid and protease-rich environment of the small intestine and also activity towards the inflammatory proline-rich prolamins from wheat, barley and rye [28, 29]. With the usage of PP1, volumetric secretion yield from *L. lactis* can be further increased 2.2-fold. As with the Mx PEP secretion in *L. casei* [7], we expect that this secretion can be further improved by utilization of pH controlled fermentation and carbon source. To eventually utilize the Fm PEP secretion for mucosal delivery of proteins, GRAS status of LAB strain and stable chromosomal integration of the gene have to be accomplished.

## Conclusion

From deposited genomics data, we identified three new naturally occurring propeptide candidates for secretion enhancement. Through characterization of these three propeptides, along with a positive control LEISSTCDA, we were able to demonstrate that these propeptides are not only comparable to LEISSTCDA as a secretion enhancement but also in the optimization of Fm PEP, they outperformed LEISSTCDA. Depending on the combination of protein of interest and propeptides, 1.4–2.3-folds increase in volumetric secretion yields were observed. In this work, we have also demonstrated, for the first time, expression and secretion of functional Fm PEP in *L. lactis*, subsequently, further optimization would be required to fully utilize the potential of *L. lactis* as a delivery and secretion vector.

## Methods

### Bacteria, LAB strains, vector and culture media

NZ9000 strain and pNZ8148 plasmid of NICE^®^ Expression System were obtained from Boca Scientific. Genes were synthesized by Integrated DNA Technologies. Growth media, M17 and GM17, were obtained from BD Biosciences (USA).

### Protein cassette construction on pNZ8148

Genes were codon-optimized using Integrated DNA Technologies’ codon optimization tool for *L. lactis cremoris*. The codon-optimized genes were amplified from synthesized Gblocks^®^ gene fragments (Integrated DNA Technologies) using the KOD-Xtreme kit (Merck). The PCR products were *Dpn*I-treated for at least 2 h and then cleaned up and concentrated using the DNA clean and concentrator kit (Zymo research). pNZ8148 were digested with restriction enzymes for at least 5 h at 37 °C. They were then incubated with thermosensitive alkaline phosphatase TSAP (Promega) for 2 h before they were cleaned and concentrated. The genes were then assembled into the vectors using Gibson assembly mix (New England Biolabs) for 1 h at 50 °C. 2 μL of the Gibson assembly mixture was added into 50 μL of electrocompetent NZ9000 cells and electroporated using 0.1 cm cuvette at 1800 V. 1 mL of GM17 media with 20 mM MgCl_2_ and 2 mM CaCl_2_ was added immediately after electroporation. The cuvette was kept on ice for 5 min before incubating the cells at 30 °C for 1–2 h. The cells were centrifuged and resuspended in 100 μL of media before they were plated out on M17 with 0.5% glucose (GM17) agar with 10 µg/mL chloramphenicol and incubated at 30 °C for 2 days. Colonies were screened for the correct construct before isolation and sequencing of the plasmids were performed.

### Protein expression and cell lysate fraction extraction

2% of overnight culture was inoculated into 50 mL of fresh GM17 media. The culture was grown statically at 30 °C to OD_600_ 0.5 before inducing with 10 ng/mL nisin. The culture, supernatant and cell pellet, was harvested 3 h after nisin induction by centrifugation at 4600 rpm for 10 min. The cell pellet was washed and re-suspended in 300 µL of Lysis Equilibration Wash buffer (LEW buffer: 50 mM NaH_2_PO_4_, 300 mM NaCl, pH 8.0). 1 mg/mL lysozyme and 50 U/mL mutanolysin were added to the cell suspensions and the cell suspensions were incubated at 30 °C for 30 min. The cell suspensions were kept on ice and sonicated 4 times for 10 s at 10 s interval using Microson XL2000 sonicator at 22.5 kHz. The cell lysate was spun down at 10,000 g for 30 min at 4 °C and the supernatant was removed as the soluble fraction. The remaining pellets were washed and re-suspended in denaturing buffer (50 mM NaH_2_PO_4_, 300 mM NaCl, 8 M Urea, pH 8.0) and spun down (10,000 g, 20 min) to obtain the insoluble fraction. Biological triplicates were performed.

### Secretion fraction

30 mL of the media fraction after 3 h of induction was removed. This was buffer exchanged and concentrated to 200 μL at 4 °C using an Amicon ultra centrifugal filter with cold 10 mM sodium phosphate buffer, pH 7.0. To ensure that cell lysis of NZ9000 strains were insignificant during production [13], we compared the genomic DNA content in culture media of intact cells to completely lysed cells at the point of harvest, using quantitative PCR with *L. lactis* specific primers for the housekeeping *tuf* gene as described [30]. Based on this comparison, cell lysis was predicted to be kept to under 0.1%.

### Immunoblotting

Protein samples were analysed on NuPAGE 4–12 or 12% Bis–Tris Gel (Life Technologies). The proteins were then transferred on to a nitrocellulose membrane using semi-dry method (Trans-Blot; Biorad) at 20 V for 20 min. The membrane was washed with PBST (PBS with 0.1% Tween) and then blocked using 5% w/v non-fat dry milk in PBST (Biorad) for 1 h at room temperature and then washed with PBST. 1:10,000 Anti-His Antibodies (Millipore) in Signal Enhancer HIKARI Solution B (Nacalai Tesque) was added to the membrane and incubated at 4 °C overnight before detecting with Clarity Western ECL Blotting Substrates (Biorad) using manufacturer’s protocol. Densitometry were performed using ImageJ. Protein yields were calculated with respect to the no-propeptide-containing-USP45 construct, using secreted proteins per culture volume. Secretion efficiencies were calculated as the secreted protein over total proteins produced.

### Z-gly-pro-4-nitroanilide assay

2 μL of the concentrated secreted fraction was added to a mixture containing 5 μL of 2 mM Z-gly-pro-4-nitroanilide (in 1,4-dioxane) and 100 μL of 10 mM sodium phosphate buffer, pH 7.0. The release of *p*-nitroanilide was measured at 410 nm using technical triplicates.

## Additional file

**Additional file 1: Figure S1.** TRX secretion. **Figure S2.** Enzymatic activity of a representative set of intracellular fractions for pNZ8148 vector only, USP45-FmPEP, USP45-PP1-, -PP2-, -PP3-, -PC-FmPEP. **Table S1.** Comparison of Fm PEP constructs.

## Abbreviations

LAB: lactic acid bacteria
GRAS: generally regarded as safe
SP: signal peptide
TRX: thioredoxin
Fm PEP: *Flavobacterium meningosepticum* prolyl endopeptidase
Mx PEP: *Myxococcus xanthus* prolyl endopeptidase
