# Supplementary material for: A propeptide toolbox for secretion optimization of Flavobacterium meningosepticum endopeptidase in Lactococcus lactis
Source: Microb Cell Fact. 2017 Dec 5;16:221. doi: 10.1186/s12934-017-0836-0 (PMC5715515; doi:10.1186/s12934-017-0836-0)
Supplement: Supplementary file 1 — Additional file 1: Figure S1. TRX secretion. Figure S2. Enzymatic activity of a representative set of intracellular fractions for pNZ8148 vector only, USP45-FmPEP, USP45-PP1-, -PP2-, -PP3-, -PC-FmPEP. Table S1. Comparison of Fm PEP constructs. [file 12934_2017_836_MOESM1_ESM.docx]

**A propeptide toolbox for secretion optimization of *Flavobacterium meningosepticum endopeptidase* in *Lactococcus lactis***

Pei Yu, Lim^1^, Lee Ling, Tan^2^, Dave Siak-Wei, Ow^1*^, Fong T., Wong^2*^

^1^ Microbial Cell Group, Bioprocessing Technology Institute, Agency for Science, Technology and Research (A*STAR), 20 Biopolis Way, #06-01 Centros, Singapore 138668

^2^ Molecular Engineering Lab, Biomedical Sciences Institutes, A*STAR, 61 Biopolis Drive, Singapore 138673

*Corresponding emails: [wongft@bmsi.a-star.edu.sg](mailto:wongft@bmsi.a-star.edu.sg), [dave_ow@bti.a-star.edu.sg](mailto:dave_ow@bti.a-star.edu.sg)

**Additional file**

**Figure S1.** TRX secretion. Cell lysate (C) and secreted (S) fractions for NZ9000 strains containing vector* (empty pNZ8148 only), constructs with TRX without USP45 SP and USP45 SP-TRX.

**Figure S2.** Enzymatic activity of a representative set of intracellular fractions for pNZ8148 vector only, USP45-FmPEP, USP45-PP1-, -PP2-, -PP3-, -PC-FmPEP. Release of p-nitroanilide, by cleavage of Z-gly-pro-4-nitroanilide, measured at 410 nm with time (seconds).

**Table S1. Comparison of Fm PEP constructs**

|  | % w.r.t. to USP45-no PP- Fm PEP (s.d.) | | |
| --- | --- | --- | --- |
|  | Enzyme activity | Volumetric protein yield | Specific protein yield |
| No PP | 100 (12) | 100 (25) | 100 (25) |
| PP1 | 226 (12) | 218 (47) | 232 (53) |
| PP2 | 201 (23) | 175 (34) | 166 (35) |
| PP3 | 205 (25) | 178 (29) | 276 (68)* |
| PC | 144 (17) | 137 (35) | 134 (34) |
| * average OD of PP3 is 1.8 compared to 2.7 for no PP | | |  |
